# Mycorrhizal symbiosis promotes the nutrient content accumulation and affects the root exudates in maize

**DOI:** 10.1186/s12870-021-03370-2

**Published:** 2022-02-05

**Authors:** Junqing Ma, Wenqi Wang, Juan Yang, Shengfeng Qin, Yisen Yang, Chenyu Sun, Gen Pei, Muhammad Zeeshan, Honglin Liao, Lu Liu, Jinghua Huang

**Affiliations:** 1 Guangxi Colleges and Universities Key Laboratory of Crop Cultivation and Tillage, College of Agriculture, Guangxi University, Nanning, 530004 Guangxi China; 2 National Demonstration Center for Experimental Plant Science Education, Guangxi University, Nanning, 530004 Guangxi China

**Keywords:** Arbuscular mycorrhizal fungi, Root vigor, Nutrient content, Organic acids

## Abstract

**Background:**

Arbuscular mycorrhizal fungi (AMF) are a group of important symbiotic microorganisms found in ecosystems. Maize is the second most produced food crop globally. To investigate the mechanisms by which mycorrhizal symbiosis improves maize yields, the effects of mycorrhizal symbiosis on root vigor, nutrient accumulation in various tissues, and root exudates were investigated. We propose the following hypothesis: The secretion of organic acids in root exudates has antagonistic or synergistic effects, which are related to the rhizosphere environment. AMF symbiosis will enhance this effect.

**Result:**

*Rhizophagus aggreatus*, *Claroideoglomus etunicatum*, and *Funneliformis mosseae* were used to inoculate maize plants separately; meanwhile, maize was inoculated with the above three fungi together for another processing. The plant tissues were sampled at five growth stages: V12 (twelve-leaf), VT (Tassel), R1 (Silking), R2 (Blister), and R4 (Dough stage). The root vigor, and nutrient content in different maize organs and organic acids in root exudates were determined in these stages. The results show that mycorrhizal symbiosis significantly improved the root vigor of maize, especially for plants inoculated with *F. mosseae*. AMF symbiosis significantly increased N, P, and K accumulation. Mixed inoculation with arbuscular mycorrhizal fungi significantly promoted the accumulation of N and K in maize. P accumulation was significantly promoted by *C. etunicatum* inoculation. Mycorrhizal symbiosis reduced the levels of protocatechuic, vanillic, citric, and ferulic acid in maize root exudates and increased the levels of p-hydroxybenzoic and caffeic acid. Except for syringic, chlorogenic and succinic acid, the levels of other organic acids in root exudates were higher in plants inoculated with *F. mosseae* than in other treatments.

**Conclusion:**

This study demonstrates that mycorrhizal symbiosis improves root vigor and promotes nutrient accumulation at various sites; in addition, mycorrhizal symbiosis affects the content of organic acids in root exudates.

**Supplementary Information:**

The online version contains supplementary material available at 10.1186/s12870-021-03370-2.

## Background

Arbuscular mycorrhizal fungi (AMF) are widely distributed in nature and can form symbionts with more than 80% of plant roots in terrestrial ecosystems [1, 2]. As an important part of the natural ecosystem, they have a great impact on plant growth and production [3]. AMF symbiosis can promote the growth of host plants, increase yields and promote nutrient uptake by host plants [4]. In cases of insufficient nutrient supply, AMF symbiosis can significantly promote host plant absorption and nutrient utilization [5]. Furthermore, AMF ensure the normal growth of plants, improve plant productivity and affect dry matter distribution [6]. Their role is more obvious under conditions of P deficiency [7]. Research has found that multiple AMF combined with colonization play a greater role in promoting host growth [8]. For example, mixed inoculation with AMF could significantly improve the nutrient uptake efficiency and biomass of host plants and significantly improve the ability of host plants to combat salt stress. Previous research has shown that AMF symbiosis can affect root exudates of host plants, improving the stress resistance and nutrient absorption capacity of host plants [9, 10].

The role of root exudates in plant growth has attracted more attention from researchers [11]. Root exudates can improve soil enzymes, and microbial activities, accelerate the decomposition of plant litter [12], and affect the absorption and utilization of nutrients by plants [13, 14]. Some studies have found that plants release more root exudates under insufficient nutrient supply to promote the absorption and utilization of nutrients [15]; plant root exudates are affected by many factors, including biological and environmental factors [16]. Previous studies have investigated whether a high aluminum content can induce plants to secrete more citric acid [17–19], which may be related to the effect of root exudates on soil properties, organic pollutant migration and activity [20–22]. These studies show that AMF symbiosis could affect the root exudates of *Artemisia annua* and thus enhance the allelopathy of *Artemisia annua*. Similarly, arbuscular mycorrhizal fungi can reduce the invasion of root-knot nematodes by changing the root exudates of their hosts [23].

Moreover, AMF symbiosis can improve insect resistance by affecting the root exudates of plants [24] and could also affect changes in phenolic acid levels in root exudates of cotton, thus improving cotton resistance to Fusarium wilt [25]. In other cases, it has been shown that AMF symbiosis could affect the root exudates of tomatoes, thus affecting the rhizosphere microbial community and improving the disease resistance of tomatoes [26].

Maize is the second most produced food crop globally and has become an economically important crop [27, 28]. AMF can induce changes in maize roots [29] and promote the absorption of nutrients in maize [30]. Studies have shown that AMF symbiosis can promote the accumulation of soluble sugar in maize roots and improve the tolerance of maize plants to salt and copper stress [31, 32]. AMF can also reduce the membrane lipid peroxidation and membrane permeability of maize, increase the accumulation of osmotic adjustment substances and antioxidant enzyme activities, reduce temperature stress damage to maize plants, improve the high-temperature tolerance of maize plants, and promote their growth [33]. AMF symbiosis can also increase the number of microorganisms and soil microbial enzymes in the maize rhizosphere [34], improving maize resistance to sheath blight [35]. It was also found that the organic acids secreted by maize roots can alleviate paraquat damage to maize [36]. Nevertheless, the effect of AMF symbiosis on root exudates of maize is rarely reported. This research aimed to investigate the effect of AMF symbiosis on the changes in maize root vigor, nutrient content and distribution, and to explore the effects of organic acids content in maize root exudates after AMF symbiosis.

## Materials and methods

### Experimental materials

A greenhouse experiment was conducted at the College of Agriculture of Guangxi University in Nanning (22°50′N, 108°17′E), China, during 2018. The maize variety Zhengda 619 (the kernel type of this kind of maize is flint corn, a common single cross hybrid that can effectively resist leaf blight, Bipolaris maydis and sheath blight) was provided by the Zhengda seed industry in Sichuan, China. Three AMFs (isolated from the soil around the roots of maize plants on farmland and obtained after 1 year of maize propagation with Zhengda 619) were used in this experiment: *Rhizophagus aggreatus*, with a spore density of 78 num g^− 1^; *Claroideoglomus etunicatum* with a spore density of 71 num g^− 1^; and *Funneliformis mosseae* with a spore density of 75 nm g^− 1^. The experimental container was a plastic flower pot with a 35 cm × 25 cm height and diameter. Sterilized river sand (the sand was washed with water three times to remove impurities and then sterilized by the damp heat method for 24 h.) was used as a substrate to grow the maize plants.

### Experimental design and methods

The maize seeds were washed three times with sterile water to remove impurities on the surface, soaked in 10% hydrogen peroxide for 1 min to remove miscellaneous bacteria on the surface of the seeds, and then washed with sterile water. The sterilized seeds were sown on filter paper in petri dishes under control conditions at 28 °C. The evenly germinated seeds were planted in pots filled with 4 kg sterilized river sand. Five treatments were designed: maize plants inoculated with *Rhizophagus aggreratus* were recorded as RA, those inoculated with *Claroideoglomus etunicatum* were recorded as CE, those inoculated with *Funneliformis mosseae* were recorded as FM, those inoculated with three species of AMF were recorded as MI, and those without AMF were recorded as CK. To set each treatment, 300 g of inoculant containing spores of *Rhizophagus aggreratus*, *Claroideoglomus etunicatum* and *Funneliformis mosseae* were added to RA, CE and FM, respectively; the above three kinds of inoculants each included 100 g for MI; sterilized sand without AMF was used as the CK treatment. In this experiment, a total of 150 pots were used. Each pot was filled with 6 maize seeds *that germinated*. When the corn plants grew two real leaves, thinning was carried out, and one plant was retained in each pot. The filtrates were collected by mixing 300 g each of the bacterial and sterilized sand together, rinsing with 5 L of sterile water, and then filtering through a 600-mesh strainer. The collected filtrates were evenly added to each pot, and 30 ml was added to each pot to ensure that the various pots had consistent microbial communities except with a difference in AMF. Each pot was irrigated with 300 ml of tap water once a week. Hogland’s nutrient solution was applied every 2 weeks from the emergence of corn plants to the 47th day and applied once a week from the 48th day to the 82nd day after emergence, applying 200 ml each time (the formula and concentration of nutrient solution are shown in the supplementary materials section).

The samples were collected during the twelve-leaf (V12, 47 days after planting), tasseling (VT, 67 days after planting), silking (R1, 74 days after planting), blister (R2, 82 days after planting), and dough (R4, 104 days after planting) stages. The plants were carefully uprooted, roots were thoroughly washed three times with distilled water to remove impurities and dried with filter paper, and samples were collected. The maize root tips were cut off to measure root vigor, and the remaining root parts were stored in 50% ethanol to determine the colonization rate of different AMF on the maize. For each treatment, another three corn plants were taken back to the laboratory, and the impurities on the roots were washed with distilled water to collect root exudates and detect the nutrient content of organs.

### Determination of mycorrhizal colonization rate

The preserved plant root was subjected to transparency, decolorization, acidification, dyeing and fading treatment. For each treatment, 20 root segments were cut and placed on slides. The morphological structure, invasion point and vesicle of mycorrhizae were observed under a light microscope (LEICA DM300, Leica Microsystems Cms GmbH, Wetzlar, Germany). The colonization rate of mycorrhizae was slightly optimized following Liu [37]. The colonization rate was determined according to the following formula:

$$\mathrm{Mycorrhizal}\ \mathrm{colonization}\ \mathrm{rate}\ \left(\%\right)=\frac{\sum \mathrm{colonization}\kern0.17em \mathrm{rate}\;1+\mathrm{colonization}\kern0.17em \mathrm{rate}\;2\dots \dots +\mathrm{colonization}\kern0.17em \mathrm{rate}\;\mathrm{n}}{\mathrm{The}\kern0.17em \mathrm{total}\kern0.17em \mathrm{number}\kern0.17em \mathrm{of}\kern0.17em \mathrm{root}\kern0.17em \mathrm{segments}\kern0.17em \mathrm{observed}}\times 100$$

### Determination of root vigor

The determination of root vigor was based on Liu’s method [38] with slight modifications. For instance, to draw a standard curve, dilutions of standard colorimetric solutions of 160 μl, 120 μl, 80 μl, 40 μl, and 20 μl were prepared and configured with 2, 3, 5-triphenyte-trazolium chloride (TTC), and the absorbance value was determined at 485 nm wavelength with methanol used as a control. Later, 0.5 g of root tip-sample was accurately weighed and transferred to a 100 ml calibration tube. A mixture of 0.4% TTC phosphoric acid buffer (1:1) was added. After 2 h of shading at 37 °C, the sample was shaken. The absorbance was measured at a wavelength of 485 nm, and the reduced amount of TTC (μg) was calculated. Root vigor was calculated as follows:

$$\mathrm{Reduction}\ \mathrm{strength}\ \mathrm{of}\ \mathrm{tetrazole}\ \left(\upmu \mathrm{g}\;{\mathrm{g}}^{-1}\;{\mathrm{h}}^{-1}\right)=\frac{\mathrm{Reduction}\kern0.17em \mathrm{of}\kern0.17em \mathrm{tetrazolium}\left(\upmu \mathrm{g}\right)}{\mathrm{Weight}\kern0.17em \mathrm{of}\kern0.17em \mathrm{root}\left(\mathrm{g}\right)\times \mathrm{Time}\left(\mathrm{h}\right)}$$

### Determination of nutrient content

The roots, stems, leaves and grains of maize at harvest were dried to a constant weight and crushed. Approximately 0.1 g of the crushed sample was placed in a digestion tube. Then, 5 ml of concentrated sulfuric acid and 2 ml of H_2_O_2_ were added and left for 24 h. The above samples were placed on a boiler for digestion until colorless and transparent, collected and then cooled at room temperature. The sample was then transferred to a 50 ml volumetric flask, serving as the determination solution. Nutrient content in different plant tissues was determined [39]. Briefly, the total nitrogen level was determined by an AutoAnalyzer 3 continuous Flow Chemical Analyzer (Bran Luebbe, Norderstedt, Germany); phosphorus was measured by a spectrophotometer (Spectrum SP-1920, Shanghai, China), and potassium was determined by a flame photometer (Aopu AP1200, Shanghai, China).

### Collection and detection of root exudates

The extraction and identification of root exudates were optimized on the basis of Badri’s method [16]. The root exudate concentrate was dissolved in chromatographic grade methanol (Fisher Scientific), passed through an organic filter membrane with a pore size of 0.22 μm (micropore), transferred to an injection bottle and stored at a low temperature.

An ultra-high-performance liquid chromatography (UPLC - QTOF; Waters Xevo G2-XS QTOF, Massachusetts, USA) chromatographic system was used to analyze the root exudates. Root exudates were separated by gradient elution on an HSS T3 column (Waters Acquity 1.8 μm, Massachusetts, USA). The mass spectrometry system included a quadrupole time flight mass analyzer (QTOF), and detection mode tandem mass spectrometry (MS/MS) was used. The specific UPLC and MS/MS conditions of each organic acid are presented in the supplementary materials section.

The content of organic acids in maize root exudates was quantitatively analyzed by Target Lynx (Waters). First, each organic acid’s quantitative ion and retention time were obtained from the standard sample, and the corresponding standard curve was drawn. The sample parameters obtained by UPLC-QTOF were brought into the standard curve to calculate the organic acid level.

Source of reagents: Standard organic acids (p-hydroxybenzoic acid, protocatechuic acid, vanillic acid, syringic acid, citric acid, p-coumaric acid, chlorogenic acid, caffeic acid, succinic acid, and ferulic acid) were purchased from Shanghai Yuanye Biotechnology Co., Ltd. (Shanghai, China).

### Statistical analysis

All of the data obtained in this experiment were calculated using Microsoft Excel 2016, and statistical analyses were carried out using SPSS (Version 19.0; IBM, Inc.) software package for Windows. Statistically significant differences among treatments were determined by one-way ANOVA and least significant difference (LSD) calculations at the 95% confidence level. Figures were mapped using the R program (https://www.r-project.org/) and SigmaPlot (Version 10.0, Systat, Inc).

## Results

### Mycorrhizal colonization rate of maize by AMF at different growth stages

**Fig. 1:**
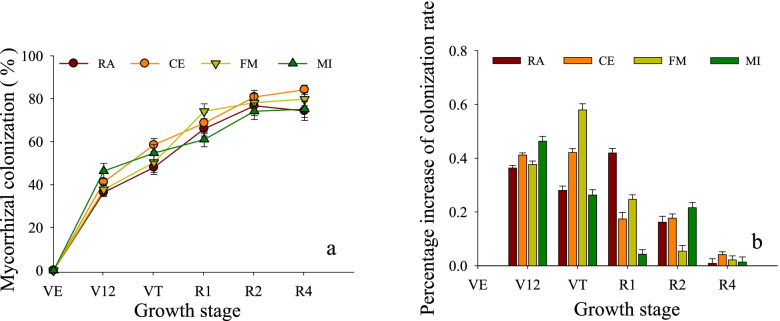
**a** Shows the changes in mycorrhizal colonization rates. **b** Shows the growth rate of the mycorrhizal customization rate V12 stands for the twelve-leaf stage (47 days after planting), VT stands for the tasseling stage (67 days after planting), R1 stands for the silking stage (74 days after planting), R2 stands for the blister stage (82 days after planting), and R4 stands for the dough stage (104 days after planting). RA denotes maize inoculated with *R. aggratus*, CE denotes maize inoculated with *C. etunicatum*, FM denotes maize inoculated with *F. mosseae*, MI denotes maize inoculated with the above three kinds of AMF, and CK denotes maize without AMF inoculation. The same applies below

The fungal colonization rate of AMF increased with the extension of the maize growth period, but the colonization rate of AMF tended to stabilize at R2 and R4 (Fig. 1). At V12, there was a significant difference between MI (46.33%) and RA (36.33%). At VT, the fungal colonization of FM on maize increased rapidly, reaching 59.50%, with an increase of 58% compared to that of V12, and the increase rate and fungal colonization were the highest recorded throughout the whole growth period. At R1, the fungal colonization of *R. aggreratus* on maize increased by 42%, representing the largest increase occurring in this period. However, in this period, the AMF with the highest colonization rate (74.17%) of maize roots was *F. mosseae.* Up to R4, the fungal colonization levels of each treatment from high to low were ranked as CE > FM > MI > RA with percent values of 84.17, 79.83, 75.17 and 74.33%, respectively.

### Root vigor of maize after symbiosis with different AMF

**Fig. 2:**
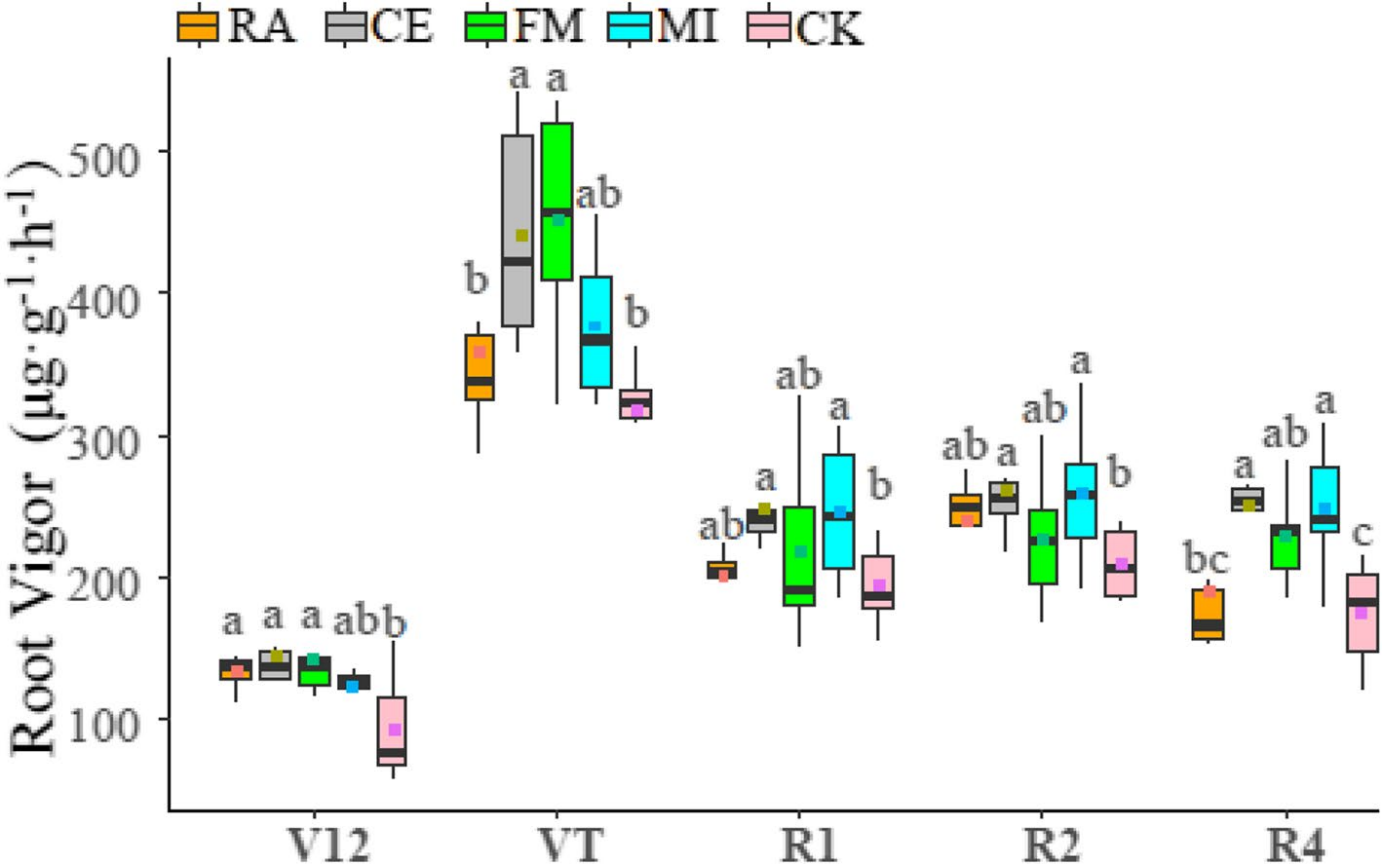
Changes in root vigor of maize under different treatments at different growth stages. Lowercase letters in the figures indicate significant differences between treatments (*p*<0.05), and for treatment and plant growth details, see the legend of Fig. 1

The root vigor of maize at different growth stages is shown in Fig. 2. In the early stages of maize growth, the root vigor of RA, CE, and FM was significantly higher than that of CK, and that of CE was the highest. The root vigor of each treatment increased rapidly and reached the maximum value at VT, and in this growth period, the root vigor levels under the RA, CE, FM, MI and CK treatments were 359.64, 442.08, 451.44, 377.28, and 317.88 μg g^− 1^ h^− 1^, respectively. At R1, the root vigor of maize decreased and maintained a relatively stable state in the later growth period; however, the root vigor of maize symbiosis with AMF was always significantly higher than that of non-AMF symbiosis. After that start of R1, the root vigor of CE was always higher than that of the other treatments and significantly higher than that of CK.

### Nutrient content of maize at different growth stages after symbiosis with different AMF

**Fig. 3:**
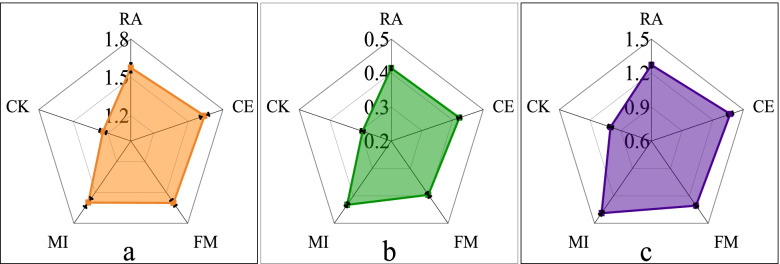
Nutrient content of maize in the harvest period. **a** Total nitrogen content of each treatment (g plant^− 1^), **b** phosphorus content in each treatment (g plant^− 1^), and **c** potassium content in each treatment (g plant^− 1^)

The total N, P and K levels of the maize plants were measured and analyzed during the harvest period. The results show that the accumulation of N, P and K in MI was the highest, and the accumulation capacity of N, P and K in maize symbiosis with AMF was significantly higher than that in CK (Fig. 3). AMF increased the accumulation of nutrients and affected the distribution of nutrients in maize organs relative to the control plants (Table 1). However, the AMF and combined treatments showed various effects on the accumulation of nutrients in the different growth stages. The effect of mycorrhizal and maize symbiosis on nutrient content was more obvious in the early and late stages than in the middle stage. For example, at V12, shortly after AMF and maize established a symbiotic relationship, the RA treatment helped maize leaves accumulate 31% more P than the CK treatment. The CE treatment accelerated the accumulation of N and K in leaves and of P and K in roots relative to the other treatments, and P accumulation in roots was 102% higher than that observed in CK. Similarly, higher N levels in roots and higher N and P levels in stems accumulated under the FM treatment in the V12 growth stage. In this regard, relative to the control, the N content in roots and P content in stems in the FM treatment increased by 114 and 157%, respectively. The nutrient contents of N, P and K in mycorrhizal symbiotic maize were significantly higher than those in AMF free maize at VT, R1 and R2. At the R4 growth stage, the dry matter accumulation of maize organs tended to be stable. After analyzing the data of this growth period, we found that the RA treatment promoted the accumulation of higher N and P in roots and more N in stems than the remaining treatment. For instance, the accumulation of N in stems was 78% higher than that in CK. Similarly, significantly higher P and K levels were found under the CE treatment in stems and roots, respectively. The accumulation of P in stems was 80% higher than that in CK. Treatment FM encouraged leaves to accumulate more N, and N accumulation was 22% higher than that under CK. The leaves of MI accumulated more P and K, and the stems accumulated more P. The accumulation of P in stems was 105% higher than that in CK. The accumulation of N, P and K in MI grains was also the highest, and P accumulation in MI grains was 109% higher than that in CK.

**Table 1:** Nutrient content in different tissues of maize under different treatments at different growth stages

Treatment	N (mg plant^−1^)				P (mg plant^− 1^)				K (mg plant^− 1^)			
	Root	Stem	Leaf	Grain	Root	Stem	Leaf	Grain	Root	Stem	Leaf	Grain
V12												
RA	381.12 ± 20.31bc	230.62 ± 17.33b	635.48 ± 20.15bc	**/**	43.29 ± 4.16bc	32.28 ± 2.05b	99.03 ± 1.77a	**/**	138.25 ± 8.48a	194.61 ± 2.48b	396.38 ± 10.99bc	**/**
CE	414.49 ± 39.26b	263.29 ± 16.71ab	727.74 ± 27.42a	**/**	54.69 ± 2.72a	33.70 ± 1.91b	94.03 ± 1.38a	**/**	154.29 ± 10.35a	214.76 ± 6.48a	493.22 ± 3.47a	**/**
FM	562.44 ± 25.27a	300.81 ± 10.95a	662.61 ± 25.37ab	**/**	44.44 ± 3.30b	50.72 ± 1.21a	98.76 ± 4.54a	**/**	151.09 ± 7.06a	218.26 ± 3.38a	410.44 ± 8.22b	**/**
MI	317.98 ± 20.95 cd	226.52 ± 15.00b	680.14 ± 28.88ab	**/**	34.36 ± 1.30 cd	38.13 ± 2.31b	95.52 ± 3.00a	**/**	99.13 ± 8.74b	225.58 ± 2.67a	385.93 ± 2.89c	**/**
CK	262.83 ± 17.87d	156.75 ± 5.61c	557.28 ± 35.55c	**/**	27.07 ± 2.69d	23.51 ± 2.59c	75.52 ± 1.99b	**/**	89.48 ± 7.89b	146.21 ± 2.36c	334.52 ± 1.68d	**/**
*P* values	0.0001	0.0003	0.0169	**/**	0.0007	0.0001	0.0007	**/**	0.0007	0.0001	0.0001	**/**
CV(±%)	11.32	9.60	7.41	**/**	12.37	10.9	4.72	**/**	12.18	2.99	2.33	**/**
VT												
RA	573.90 ± 28.86b	273.94 ± 10.45a	861.43 ± 27.44a	**/**	60.44 ± 3.09b	105.82 ± 5.63a	169.74 ± 6.96a	**/**	182.85 ± 7.34a	318.75 ± 4.84a	620.27 ± 19.27c	**/**
CE	659.7419.94a	236.19 ± 5.98b	829.00 ± 40.04a	**/**	75.95 ± 2.25a	96.39 ± 4.21ab	141.65 ± 5.94b	**/**	198.48 ± 10.55a	303.71 ± 12.20a	870.70 ± 6.66a	**/**
FM	645.69 ± 18.38a	273.25 ± 10.54a	816.58 ± 38.92ab	**/**	64.23 ± 1.35b	86.64 ± 4.47bc	137.61 ± 3.24b	**/**	135.66 ± 3.45b	262.82 ± 7.75b	801.25 ± 1.73b	**/**
MI	641.33 ± 11.75a	264.39 ± 11.95ab	797.57 ± 40.43ab	**/**	79.69 ± 1.99a	85.97 ± 4.04bc	126.11 ± 6.39b	**/**	184.57 ± 7.27a	315.55 ± 9.35a	868.46 ± 11.44a	**/**
CK	431.60 ± 23.46c	255.88 ± 16.56ab	717.59 ± 24.55ab	**/**	60.49 ± 2.08b	79.00 ± 3.27c	105.49 ± 8.12c	**/**	134.88 ± 11.16b	247.61 ± 10.11b	632.22 ± 17.03c	**/**
*P* values	0.0001	0.2050	0.1223	**/**	0.0002	0.0120	0.0005	**/**	0.0006	0.0007	0.0001	**/**
CV(±%)	6.29	7.34	7.34	**/**	5.58	8.2	8.11	**/**	8.34	5.38	2.80	**/**
R1												
RA	579.79 ± 41.04a	207.77 ± 10.05bc	672.99 ± 6.13a	**/**	52.30 ± 3.39a	84.65 ± 1.93c	133.31 ± 4.17a	**/**	139.73 ± 1.83b	228.52 ± 2.84b	540.05 ± 7.89a	**/**
CE	590.17 ± 7.38a	238.35 ± 10.34ab	605.41 ± 20.86ab	**/**	54.15 ± 5.37a	114.42 ± 3.12a	134.24 ± 7.39a	**/**	154.56 ± 4.88b	227.85 ± 1.67b	483.31 ± 10.75b	**/**
FM	552.94 ± 14.29a	231.30 ± 12.95ab	613.44 ± 32.51ab	**/**	51.50 ± 1.36a	99.11 ± 4.00b	101.41 ± 3.83b	**/**	216.11 ± 6.46a	235.80 ± 3.94b	455.60 ± 9.23c	**/**
MI	320.29 ± 6.83b	263.18 ± 11.81a	600.28 ± 29.69ab	**/**	45.38 ± 1.13a	116.92 ± 4.33a	137.21 ± 5.96a	**/**	145.00 ± 2.15b	318.04 ± 10.06a	502.60 ± 3.03b	**/**
CK	309.49 ± 9.97b	193.44 ± 10.69c	571.83 ± 18.80b	**/**	47.88 ± 1.15a	69.87 ± 3.63d	103.04 ± 4.43b	**/**	137.50 ± 9.93b	188.12 ± 8.84c	407.33 ± 5.67d	**/**
*P* values	0.0001	0.0060	0.1100	**/**	0.3008	0.0001	0.0010	**/**	0.0001	0.0001	0.0001	**/**
CV(±%)	5.63	7.09	6.20	**/**	8.28	6.21	7.29	**/**	5.60	3.99	2.67	**/**
R2												
RA	363.93 ± 20.82a	396.64 ± 14.96d	453.24 ± 27.72c	**/**	66.09 ± 4.18a	333.52 ± 4.55b	125.73 ± 4.10b	**/**	292.66 ± 9.16b	648.10 ± 3.98b	552.82 ± 4.14a	**/**
CE	297.65 ± 10.88bc	884.62 ± 33.42a	506.31 ± 24.44c	**/**	61.33 ± 2.81ab	314.23 ± 5.21bc	98.55 ± 3.98c	**/**	295.55 ± 3.39b	651.59 ± 2.57b	495.71 ± 4.93c	**/**
FM	272.98 ± 15.69c	627.30 ± 27.67c	591.88 ± 13.49b	**/**	55.20 ± 3.39b	300.70 ± 6.31c	107.42 ± 2.53c	**/**	328.90 ± 5.03a	579.48 ± 4.70c	491.66 ± 2.02c	**/**
MI	314.27 ± 4.98b	796.67 ± 19.09b	776.05 ± 42.63a	**/**	58.02 ± 1.26ab	390.85 ± 11.38a	137.77 ± 7.06a	**/**	248.46 ± 5.27c	676.66 ± 7.25a	532.75 ± 2.29b	**/**
CK	285.39 ± 3.81bc	465.13 ± 27.74d	447.00 ± 16.54c	**/**	53.31 ± 4.12b	214.76 ± 9.68d	98.52 ± 5.81c	**/**	164.60 ± 13.92d	466.76 ± 6.83d	394.05 ± 5.98d	**/**
*P* values	0.0047	0.0001	0.0001	**/**	0.1268	0.0001	0.0001	**/**	0.0001	0.0001	0.0001	**/**
CV(±%)	6.25	7.04	7.76	**/**	9.33	4.34	3.24	**/**	5.67	1.51	1.42	**/**
R4												
RA	460.18 ± 17.27a	492.49 ± 15.83a	325.48 ± 9.38b	669.94 ± 35.73c	53.10 ± 2.52a	363.28 ± 4.48c	120.15 ± 4.82b	230.58 ± 4.24b	249.72 ± 7.85b	1263.62 ± 23.14ab	460.57 ± 11.13d	167.36 ± 1.98c
CE	429.83 ± 21.30ab	434.38 ± 35.33ab	316.19 ± 24.83b	769.54 ± 13.67b	50.62 ± 0.13a	447.22 ± 6.36a	93.68 ± 9.63bc	246.00 ± 6.02a	267.81 ± 4.32a	1283.46 ± 33.51ab	549.96 ± 8.42c	197.50 ± 5.86b
FM	347.36 ± 32.14c	375.62 ± 40.90b	397.27 ± 18.66a	734.79 ± 20.12bc	51.24 ± 3.14a	392.35 ± 8.50b	167.65 ± 14.44a	171.80 ± 1.54c	230.85 ± 4.11c	1216.85 ± 19.95b	611.02 ± 5.28b	184.30 ± 6.39b
MI	364.75 ± 17.23bc	360.42 ± 13.87bc	352.82 ± 7.17ab	913.40 ± 24.23a	50.59 ± 1.93a	325.32 ± 7.56d	183.69 ± 6.45a	255.76 ± 4.85a	258.92 ± 6.41ab	1318.86 ± 24.13a	637.11 ± 2.78a	224.27 ± 6.13a
CK	387.22 ± 11.33bc	276.95 ± 14.79c	326.69 ± 22.78b	563.87 ± 23.25d	47.50 ± 2.21a	248.60 ± 12.15e	89.40 ± 6.52c	122.57 ± 2.84d	203.37 ± 4.13d	931.98 ± 9.84c	395.37 ± 2.94e	124.443.45d
*P* values	0.0194	0.0022	0.0557	0.0001	0.5419	0.0001	0.0001	0.0001	0.0001	0.0001	0.0001	0.0001
CV(±%)	8.92	10.88	8.47	5.76	6.78	4.17	11.67	3.26	3.27	3.11	1.60	4.55

The five parts in Table 1 are the five growth stages of maize, which are V12 (twelve-leaf stage), VT (tasseling stage), R1 (silking stage), R2 (blister stage), and R4 (dough stage stage). Each growth stage included five treatments, namely RA (maize inoculated with *R. aggratus*), CE (maize inoculated with *C. etunicatum*), FM (maize inoculated with *F. mosseae*), MI (maize inoculated with the above three kinds of AMF), and CK (maize without AMF inoculation). Lower case letters in figures differently indicate significant differences between treatments (*p*<0.05)

### Changes in organic acids in maize root exudates at different growth stages after symbiosis with AMF

**Fig. 4:**
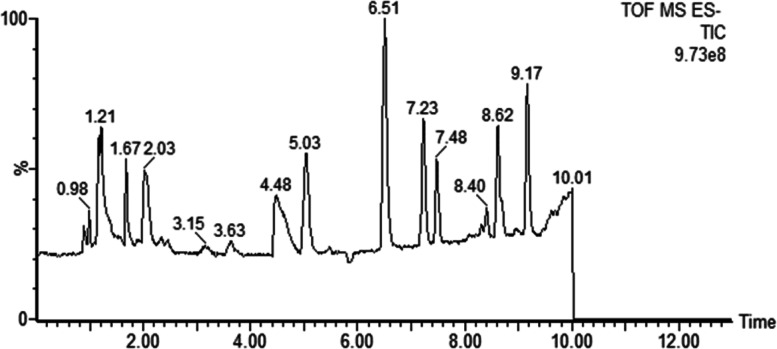
Primary chromatograms of 10 organic acids standards

**Fig. 5:**
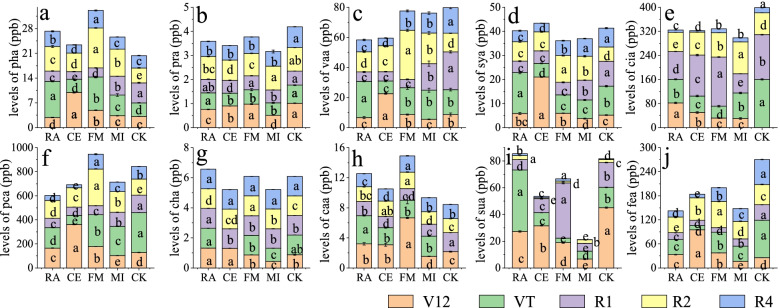
Organic acids in maize root exudates under different AMF symbiosis levels (ppb). (a ~ j) show the differences in p-hydroxybenzoic acid, protocatechuic acid, vanillic acid, syringic acid, citric acid, p-coumaric acid, chlorogenic acid, caffeic acid, succinic acid, and ferulic acid secretion in different growth stages under different treatments. Different colored blocks in each stacked histogram represent different growth maize periods: orange blocks refer to R4 (dough stage), green blocks refer to R2 (blister), purple blocks refer to R1 (silking), yellow blocks refer to VT (tassel), and blue blocks refer to V12 (twelve leaf). Analysis of differences between groups was performed for different treatments of the same growth phase shown in each stained histogram with identical lowercase letters denoting no significant differences between treatments (*P* > 0.05) and with different lowercase letters denoting significant differences between treatments (*P* < 0.05). Lower case letters in figures differently indicate significant differences between treatments (*p*<0.05)

The primary chromatograms of 10 organic acids standards are shown in Fig. 4. Different AMF inoculations had different effects on maize root exudates at different growth stages (Fig. 5). In this experiment, 10 kinds of organic acids were isolated from maize root exudates. At the V12 growth stage, syringic acid under treatment CE was 304.82% higher than that of CK, and succinic acid under treatment MI was 85.23% lower than that of CK. At the VT stage, the p-hydroxybenzoic acid levels were 166.40% higher under the RA treatment than under the CK treatment, but the secretion of ferulic acid was inhibited by *C. etunicatum*, and the ferulic acid level was 88.38% lower under the CE treatment than under the CK treatment. Similarly, in the R1 stage, the ferulic acid level was 68.96% lower upon the imposition of FM than without AMF treatment, and caffeic acid levels under CE treatment were 41.27% lower than those of CK. However, across the whole growth period, the total amount of caffeic acid in FM was significantly higher than that in all other treatments, including CK. The succinic acid content of treatment MI was 68.95% lower than that of CK at the R1 stage. At the R2 stage, FM had 168.81% more p-hydroxybenzoic acid than the CK treatment. The FM treatment promoted the secretion of p-hydroxybenzoic and p-coumaric acid at the R4 growth stage, with levels 41.71% higher than those of CK, and the succinic acid of MI was 66.07% lower than that of CK. Interestingly, regardless of the growth period, the succinic acid content of MI was the lowest across all treatments.

### Correlation analysis of root exudates and nutrient allocation

**Fig. 6:**
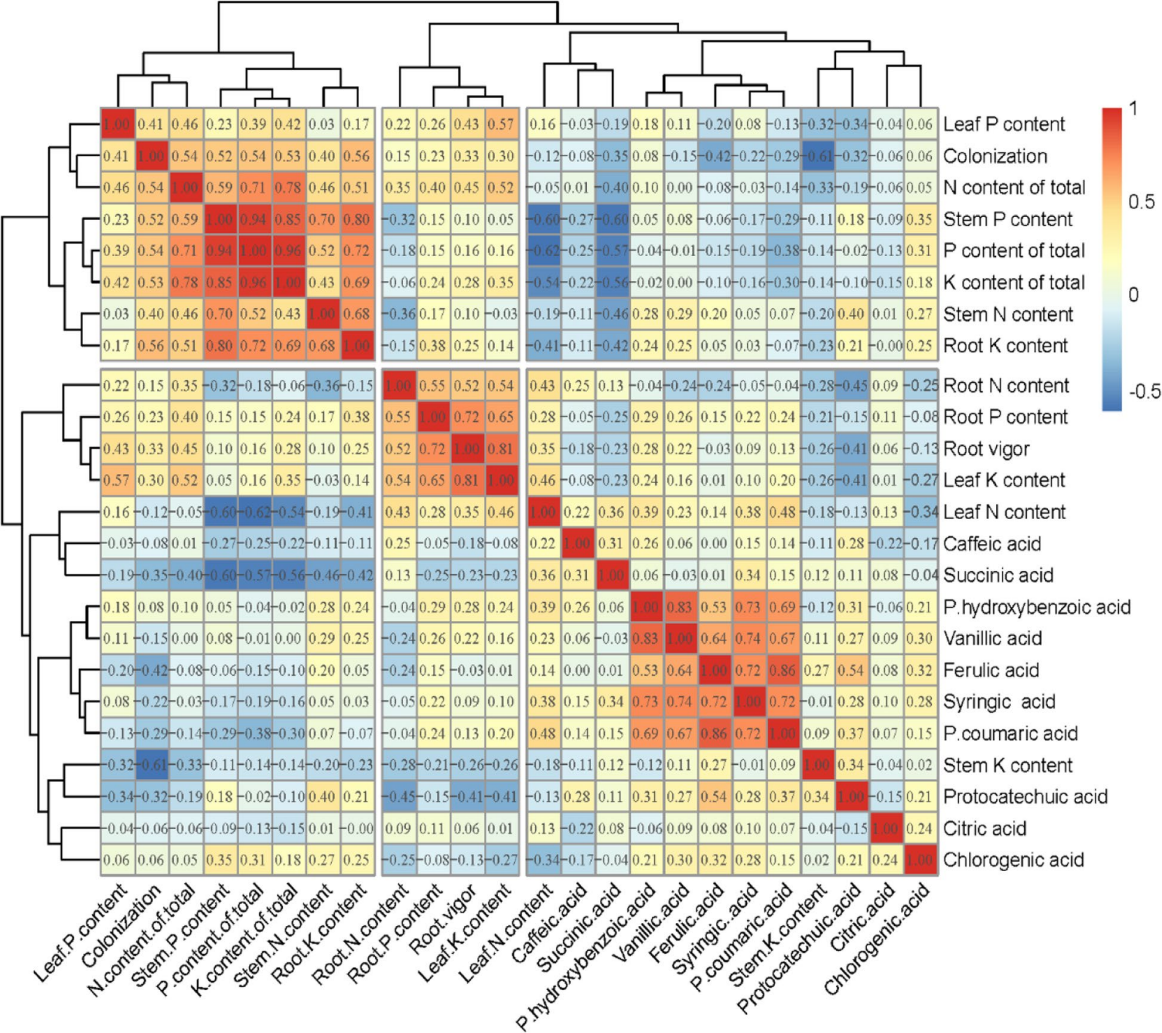
Correlation clustering heatmap of the correlation analysis. The Pearson coefficient is obtained by analyzing the correlation between each index. The obtained Pearson coefficient is made into a correlation heatmap. Red fields denote a positive correlation between the two. The darker the color is, the stronger the correlation is. Blue fields denote the opposite result

To obtain an overall understanding of the effects of AMF symbiosis on maize root vigor, nutrient accumulation and organic acids in root exudates, we drew a cluster correlation heatmap for the obtained data, and the results are shown in Fig. 6. The results show that AMF symbiosis could improve the root vigor of maize, inhibit the levels of protocatechuic and ferulic acid in root exudates, increase levels of p-hydroxybenzoic and caffeic acid, and promote nutrient accumulation in various organs. The correlation coefficients between total nitrogen and total phosphorus content and mycorrhizal infection rates were the largest at 0.54. Root vigor was positively correlated with maize nutrient accumulation and had the greatest effect on leaf potassium content, and its Pearson correlation coefficient reached 0.81. Among the organic acids, ferulic acid generated the largest Pearson correlation coefficient with the mycorrhizal infection rate, and its Pearson coefficient reached − 0.42, indicating that mycorrhizal symbiosis significantly reduces the content of ferulic acid. The clustering results show that the mycorrhizal infection rate and total nitrogen content of maize belonged to the same cluster and were positively correlated, and the Pearson correlation coefficient was 0.54. Some rules of the clustering results of organic acids apply. Citric and chlorogenic acid belong to the same cluster, and their correlation coefficient is 0.24; butyric and p-coumaric acid belong to the same cluster, and their correlation coefficient is 0.72; P-hydroxybenzoic and vanillic acid belong to the same cluster, and their correlation coefficient is 0.83; caffeic and ferulic acid belong to the same cluster, and their correlation coefficient is 0.31.

## Discussion

AMF can affect the growth, development and reproduction of host plants by regulating the absorption and recycling of nutrients. AMF symbiosis significantly increased the nutrient content of maize roots, stems and leaves, showing that AMF symbiosis could promote the nutrient content of host plants, which is consistent with the results of Chaudhary Year [4]. In addition, this study found that AMF symbiosis affects nutrient allocation in maize organs. The results show that *R. aggrratus* accumulated more N in maize leaves during VT. *C. etunicatum* caused maize roots to accumulate more N in the VT period and caused stems to accumulate more N in the R2 period and more P in the R4 period. *F. mosseae* caused maize roots to accumulate more K in the R2 period, roots to accumulated more P in the VT period, leaves to accumulated more P in the R4 period, and stems and leaves to accumulated more K in the R4 and VT periods, respectively. In all treatments, the joint action of the three AMFs most significantly promoted the accumulation of N, P and K in maize grains. AMF symbiosis significantly promoted maize nutrient accumulation, which is consistent with the research results of Chaudhary [4].

The root vigor of maize was higher at VT than at other growth periods. In this period, root vigor levels from high to low were ranked as follows: FM > CE > Mi > RA > CK. The root vigor of maize was affected by several factors, such as nutrient stress levels and cultivation methods used [15, 38]. AMF symbiosis improves plant root vigor under salt stress [40]. This study found that AMF symbiosis can still affect the root vigor of maize even without salt stress, and the root vigor of maize with AMF symbiosis was significantly higher than that of maize without AMF symbiosis in each growth stage. In addition, different AMFs have different effects on maize root vigor.

Our current study only explored changes in some organic acids in corn root exudates after AMF symbiosis. From the total level of organic acids secretion in the whole maize growth period, we found that FM promoted the secretion of p-hydroxybenzoic, p-coumaric and caffeic acid. CE promoted the secretion of syringic acid, and RA promoted the secretion of chlorogenic and succinic acid. The levels of protocatechuic, vanillic, citric and ferulic acid were lower than those of CK. Some studies have shown that p-hydroxybenzoic acid can reduce the number of *Fusarium* and the occurrence of Fusarium wilt [41]; vanillic acid can inhibit soil-borne pathogens and reduce soil-borne diseases [42]. Vanillic acid can inhibit the growth of Fusarium wilt [43]; caffeic acid plays an important role in inhibiting soil-borne pathogens, and caffeic acid can directly inhibit the growth of *Ralstonia solanacearum* [44]. AMF may alleviate soil-borne diseases [45] by increasing the levels of p-hydroxybenzoic, vanillic and caffeic acid in plant root exudates. In addition, AMF symbiosis promotes the secretion of syringic acid and inhibits the secretion of ferulic acid. Syringic acid can change the microbial community in the rhizosphere and the structure of bacterial and fungal communities in the rhizosphere [46]. Ferulic acid has strong allelopathy and can inhibit the growth of plant roots [47]. It was found that a change in plant root exudates affects the activity of rhizosphere soil microorganisms and then the composition of the microbial community [48, 49], which may be related to the promotion of syringic acid secretion by AMF symbiosis. Moreover, AMF symbiosis alleviates the continuous cropping obstacle [50], which may be related to a decrease in ferulic acid secretion. The effect of AMF symbiosis on organic acids in root exudates may be an important means through which AMF improves host plant resistance, alleviates continuous cropping barriers and promotes nutrient uptake. In general, the secretion of organic acids varied with different maize growth stages. AMF symbiosis further affected the content of organic acids in maize root exudates, and this effect was related to the type of AMF involved and the maize growth period. However, the role of these organic acids in maize growth and their impact on maize growth after AMF symbiosis must be further studied and discussed, potentially through exogenous application. In subsequent experiments, we will also further study and verify the effects of these organic acids on maize. From Fig. 6, we found that after mycorrhizal symbiosis, maize root vigor and nutrient accumulation were promoted synchronously, showing that the improvement of maize root vigor after mycorrhizal symbiosis may be an important reason behind the improvement in nutrient accumulation. Therefore, it is speculated that after mycorrhizal symbiosis, mycorrhizal symbiosis promotes the capacity for nutrient uptake and then the accumulation of nutrients in maize by enhancing root viability. From our cluster analysis results, we conclude that the content of organic acids was closely related to leaf N content and that root vigor was closely related to root N and P levels and leaf K content, whereas the mycorrhizal infection rate and total N, P, and K levels of maize plants, leaf P content, stem N content, and root K content were related. Some rules govern organic acids content in root exudates of maize, as p-hydroxybenzoic, vanillic, ferulic, symbolic, p-coumaric, cash and succinic acid did not increase synchronously; there was also a negative correlation between p-hydroxybenzoic, protocatechuic and citric acid. We know that antagonistic or synergistic effects occur between endogenous plant hormones [51]. Based on previous studies and our results, we speculate that there should also be antagonism or synergy between organic acids in root exudates closely related to the plant rhizosphere environment.

## Conclusion

Our results suggest that different mycorrhizal fungi play different roles after symbiosis with maize. *R. aggreratus* promotes the accumulation of N and P in maize roots and of N in stems. *C. etunicatum* encourages roots to accumulate more K and stems to accumulate more P. *F. mosseae* was found to have the greatest effect on the root vigor of maize and encouraged leaves to accumulate more N. The three AMF treatments promoted the accumulation of more K in stems and promoted the accumulation of more P and K in leaves, while promoting the accumulation of more N, P, and K in grains. Different mycorrhizae have different effects on root exudates. *R. aggreratus* had the greatest effect on maize root exudates at the VT growth stage, *C. etunicatum* had the greatest effect on maize root exudates at the V12 growth stage, and *F. mosseae* had the greatest effect on maize root exudates at the R1 and R2 growth stages.

We conclude that AMF symbiosis could significantly improve root vigor, promote the accumulation of nutrients, and affect nutrient distribution, and these changes are significantly correlated with changes in root exudates. The results of this study can preliminarily explain the relationship between root exudates after AMF symbiosis and provide a theoretical basis for the mechanism of improving maize yield after AMF symbiosis. The presented results also provide a theoretical basis for research on AMF use in microbial fertilizers.

## Supplementary Information

**Additional file 1.**

## Data Availability

The datasets used and analysed during the current study available from the corresponding author on reasonable request.

## Abbreviations

V12: Twelve-leaf stage (47 days after planting)
VT: Tasseling stage (67 days after planting)
R1: Silking stage (74 days after planting)
R2: Blister stage (82 days after planting)
R4: Dough stage (104 days after planting).
RA: Maize plants inoculated with *Rhizophagus aggreratus*
CE: Maize plants inoculated with *Claroideoglomus etunicatum*
FM: Maize plants inoculated with *Funneliformis mosseae*
MI: Maize plants inoculated with *Rhizophagus aggreratus*, *Claroideoglomus etunicatum* and *Funneliformis mosseae*
CK: Maize plants without AMF
